# Impact of intraoperative nonsteroidal anti-inflammatory drugs on acute kidney injury after major noncardiac surgery: a propensity score-matched analysis

**DOI:** 10.1080/0886022X.2026.2663247

**Published:** 2026-05-05

**Authors:** Cong Zhang, Yixin Liao, Xiaoyu Zhu, Wei Wang, Xinxin Liao

**Affiliations:** a State Key Laboratory of Oral & Maxillofacial Reconstruction and Regeneration, National Clinical Research Center for Oral Diseases, Shaanxi Engineering Research Center for Dental Materials and Advanced Manufacture, Department of Anesthesiology, School of Stomatology, The Fourth Military Medical University, Xi’an, Shaanxi, China; b Department of Obstetrics and Gynecology, Centre for Reproductive Medicine, Nanfang Hospital, Southern Medical University, Guangzhou, Guangdong, China; c Department of Anesthesiology, Guangdong Provincial Key Laboratory of Precision Anesthesia and Perioperative Organ Protection, Nanfang Hospital, Southern Medical University, Guangzhou, Guangdong, China

**Keywords:** Acute kidney injury, NSAIDs, propensity score matching, major noncardiac surgery

## Abstract

Nonsteroidal anti-inflammatory drugs (NSAIDs) are widely used as part of multimodal analgesia, but their association with acute kidney injury (AKI) in patients undergoing elective surgery remains uncertain. We conducted a retrospective, propensity score-matched study at a single-center academic hospital (ChiCTR2300076725). The primary outcome was the incidence of AKI, defined by the Kidney Disease: Improving Global Outcomes (KDIGO) serum creatinine criteria, compared between patients who did and did not receive NSAIDs during surgery. Secondary outcomes included AKI severity staging and prolonged postoperative hospitalization (≥7 days). A total of 11,139 patients were included, with 3,361 in the NSAIDs group and 7,778 in the control group. After propensity score matching (PSM), 3,361 matched pairs were obtained. The incidence of postoperative AKI was comparable between the NSAIDs and control groups after matching (4.2% *vs.* 4.2%, *p* > 0.999). The distribution of AKI stages (*p* = 0.830) and the median duration of postoperative hospital stay were also comparable between the two groups (both 6 days, *p* = 0.290). Sequentially adjusted logistic regression models consistently showed no significant association between NSAIDs and AKI, AKI staging, or prolonged hospitalization, both before and after PSM. Stratified analyses by NSAID type and dose-response analyses revealed no significant association with AKI. A sensitivity analysis using complete cases without imputation (*n* = 9,385) yielded consistent results. In conclusion, intraoperative NSAIDs administration was not independently associated with an increased risk of postoperative AKI in patients undergoing major noncardiac surgery.

## Introduction

Perioperative analgesic management plays a pivotal role in mitigating postoperative inflammation and stress responses following major surgeries. As an integral component of multimodal analgesia, nonsteroidal anti-inflammatory drugs (NSAIDs) are recommended for postoperative pain control [1]. However, their administration may potentially increase the risk of renal dysfunction [2]. Intravenous formulations of flurbiprofen axetil and parecoxib sodium exhibit potent analgesic, antipyretic, and anti-inflammatory properties, making them widely adopted in clinical practice [3].

Acute kidney injury (AKI) is a serious postoperative complication after major surgery with a reported incidence of 3–35% [4,5]. It is associated with prolonged hospital length of stay, increased costs, and higher mortality rates [6–8]. NSAIDs-associated AKI has been well-documented in several population-based studies, including those involving critically ill patients [9–11]. However, the specific impact of perioperative NSAIDs on postoperative renal function in surgical patients remains controversial, as recent evidence yields conflicting results [12]. A secondary analysis of the RELIEF trial focusing on major abdominal surgery demonstrated that intraoperative NSAIDs were associated with increased odds of a higher maximum AKI stage [13]. In contrast, other studies suggest a neutral or even potentially protective role: a prospective cohort study found no significant association between early postoperative NSAIDs exposure and AKI in patients undergoing major gastrointestinal surgery [14]. Additionally, retrospective analyses have reported that perioperative flurbiprofen axetil was associated with a reduced risk of AKI in spinal surgery patients [15] and that parecoxib conferred a modest reduction in AKI risk among patients undergoing noncardiac surgery [16].

Optimizing perioperative analgesia may help prevent postoperative complications, including AKI, although the optimal strategy remains debated. Accordingly, we conducted a retrospective cohort study to investigate the association between intraoperative NSAIDs and AKI in patients undergoing elective major noncardiac surgery. Propensity score matching (PSM) was used to ensure the robustness of the findings.

## Methods

The study protocol was reviewed and approved by the Institutional Review Board of Nanfang Hospital (NFEC-2023-275). The study was conducted in accordance with the principles of the Declaration of Helsinki. Given the retrospective design and the use of anonymized clinical data, the requirement for written informed consent was waived by the IRB. The Strengthening the Reporting of Observational Studies in Epidemiology (STROBE) guidelines were followed in study design and conduct.

### Study population

**Figure 1.**
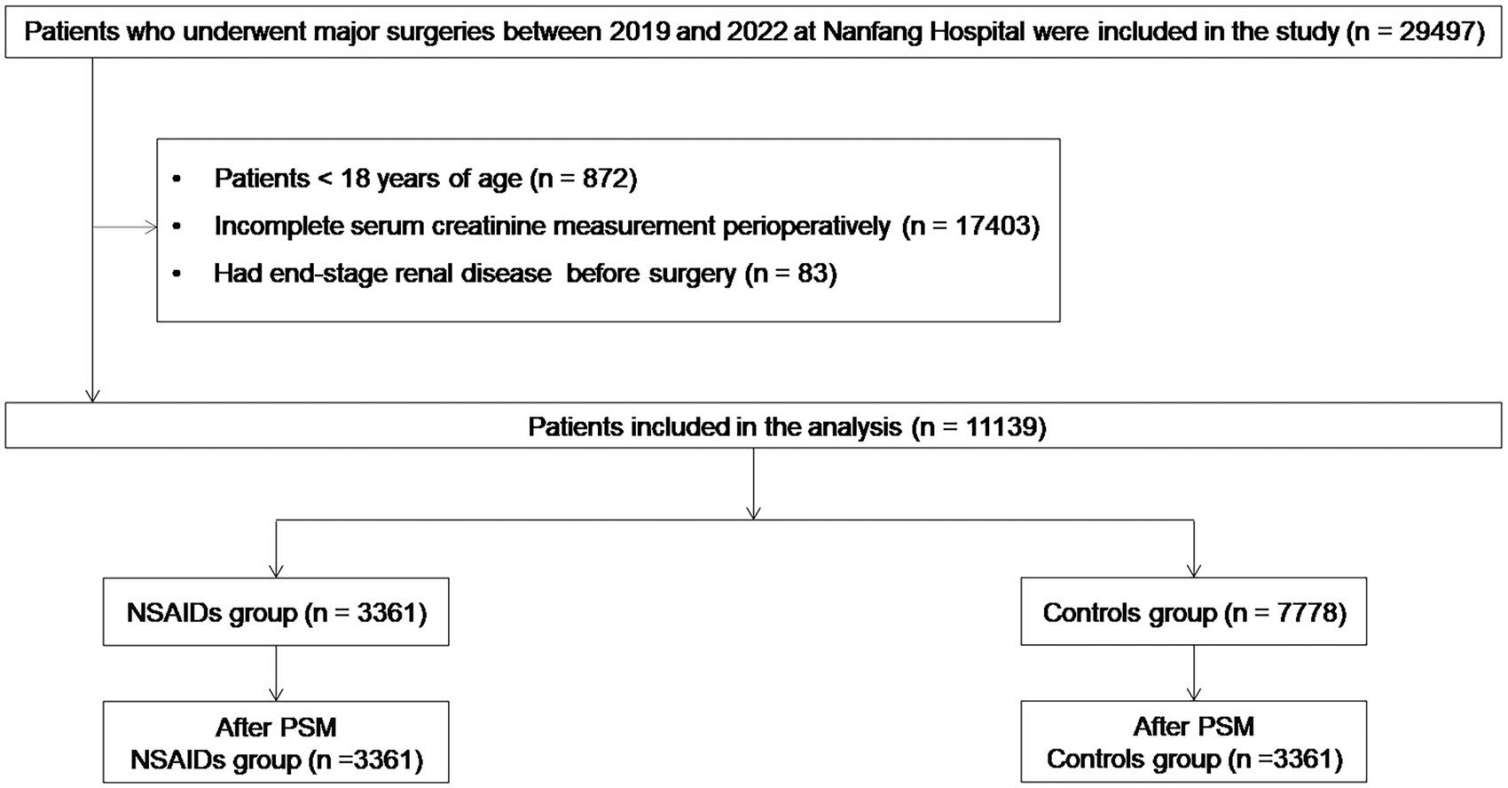
Flowchart of patient selection. A total of 29,497 patients were screened, and 11,139 were included after applying exclusion criteria. After propensity score matching, 3,361 matched pairs (6,722 patients) were analyzed. NSAIDs: nonsteroidal anti-inflammatory drugs; eGFR: estimated glomerular filtration rate.

This is a retrospective cohort study in which medical records of patients who underwent major noncardiac elective surgery at Nanfang Hospital between January 2019 and October 2022 were reviewed. Major surgery was defined as grade II or grade III surgery according to Johns Hopkins criteria (Supplementary Table S1) [17], excluding cardiovascular, organ transplantation, intracranial, urological procedures such as the relief from urinary obstruction and nephrectomy. The surgical type was determined based on the International Classification of Diseases, Ninth Revision, Clinical Modification (ICD-9-CM) procedure codes (Supplementary Table S2). In the NSAIDs group, patients received either flurbiprofen axetil or parecoxib sodium during surgery. The maximum intraoperative dose was 100 mg for flurbiprofen axetil and 80 mg for parecoxib sodium; patients who exceeded these doses were excluded. Exclusion criteria included patients under 18 years of age, those with incomplete serum creatinine measurements before or after surgery, and those with end-stage renal disease (defined as receiving preoperative renal replacement therapy or estimated glomerular filtration rate [eGFR] < 15 mL·min^−1^·1.73 m^−2^) [18] (Figure 1).

### Exposure factor and covariates

The NSAIDs administered in this study included parecoxib sodium (a selective cyclooxygenase-2 [COX-2] inhibitor) and flurbiprofen axetil (a nonselective COX inhibitor). Electronic medical records were reviewed to collect variables potentially associated with postoperative AKI. The clinical data included baseline demographics (age, sex, body mass index [BMI], and American Society of Anesthesiologists [ASA] physical status), preoperative comorbidities (hypertension, diabetes mellitus, coronary artery disease, chronic heart failure, stroke, chronic obstructive pulmonary disease [COPD], and vascular disease), preoperative medications (angiotensin-converting enzyme inhibitors [ACEI]/angiotensin receptor blockers [ARB], statins, insulin and diuretics), laboratory tests (serum creatinine, albumin, and hemoglobin), and intraoperative data (operative site, operative duration, anesthetic approaches, anesthetic duration, blood pressure, transfusion, blood loss, and fluid balance). Intraoperative hypotension (IOH) was defined as a mean arterial pressure (MAP) <65 mmHg during the surgery [19]. Decreased preoperative renal function was defined as a preoperative serum creatinine level >1.2 mg·dL^−1^. This fixed threshold has been identified as a significant predictor of postoperative acute kidney injury in both cardiac and noncardiac surgical populations, and is widely used in perioperative literature, allowing direct comparison with prior studies [20]. Hypoalbuminemia was defined as a preoperative serum albumin level <3.5 g·dL^−1^ [21]. Preoperative anemia was defined as a serum hemoglobin <100 g·L^−1^; this threshold corresponds to moderate anemia and was selected because it has been associated with adverse perioperative outcomes in surgical populations, rather than the standard World Health Organization (WHO) definitions, which were designed for general population screening [22,23].

### Outcomes

The primary outcome of this study was the incidence of AKI within 7 days after surgery, defined according to the Kidney Disease: Improving Global Outcomes (KDIGO) criteria based on serum creatinine. AKI was diagnosed as an increase in serum creatinine (SCr) by ≥0.3 mg/dL (26.5 μmol/L) within 48 h or an increase to ≥1.5 times baseline within 7 days. AKI was staged as follows: stage 1, SCr ≥0.3 mg/dL within 48 h or 1.5–1.9 times baseline; stage 2, 2.0–2.9 times baseline; stage 3, ≥3.0 times baseline or SCr ≥4.0 mg/dL (353.6 μmol/L), or initiation of renal replacement therapy, all within 7 days [24]. Urine output criteria were not used because reliable hourly urine output data were not available in the electronic medical records, and our previous study indicated that intraoperative oliguria has limited predictive value for postoperative AKI [25]. The secondary outcomes were the stage of AKI and prolonged postoperative hospital stay, defined as 7 days or more between surgery and discharge.

### Statistical analyses

To minimize the potential impact of confounding factors, propensity score (PS) matching was performed. A propensity score was calculated using logistic regression, and patients in the NSAIDs group were matched with those in the control group at a 1:1 ratio using the nearest-neighbor method. A caliper of 0.02 on the propensity score scale was applied to ensure close matching. The matching covariates included patient demographics, preoperative comorbidities and medications, preoperative laboratory tests, ASA physical status, operative site, anesthetic approaches, blood loss, transfusion requirements, duration of surgery and anesthesia, IOH, and fluid balance. The balance of baseline covariates was assessed using the standardized mean difference (SMD); values <10% were considered to indicate negligible imbalance [26].

Missing data were presented and imputed using a random forest algorithm (missRanger package in R) before PS matching. To assess the robustness of the imputation, a sensitivity analysis was conducted using the complete-case dataset, repeating the PS matching and primary regression analyses.

Continuous variables were tested for normality using the Kolmogorov-Smirnov test and were presented as median (interquartile range [IQR]). Differences between groups were compared using the Mann-Whitney *U* test. Categorical variables were presented as frequency and percentage and were compared using either the Chi-square or Fisher’s exact test, as appropriate. Binary logistic regression models were used to assess the association between NSAIDs and postoperative AKI. Ordinal logistic regression models were used to examine the relationship between NSAIDs and the stage of AKI. For prolonged postoperative hospital stay, binary logistic regression models were used. All models were sequentially adjusted: Model 1, unadjusted; Model 2, adjusted for age, sex, and BMI; Model 3, Model 2 plus coexisting medical conditions, preoperative medications, and baseline laboratory values; Model 4, Model 3 plus ASA physical status, operative site, anesthetic approaches, and intraoperative variables.

To address the concern that different NSAID types may have distinct nephrotoxic profiles, additional analyses were performed stratifying patients by NSAID type, with the control group as the reference. A dose-response analysis was conducted by categorizing NSAID doses into low-dose (parecoxib ≤40 mg or flurbiprofen axetil ≤50 mg) and high-dose groups (parecoxib >40 mg or flurbiprofen axetil >50 mg).

Subgroup analyses were performed to investigate the association between NSAIDs and postoperative AKI in predefined subgroups, including patients with or without preoperative hypoalbuminemia, decreased or normal preoperative renal function, with or without IOH, and with or without preoperative anemia, using binary logistic regression models with full covariate adjustment. The primary subgroup analyses were conducted on the propensity score-matched cohort to minimize residual confounding. To assess consistency and maximize statistical power, the same analyses were repeated on the entire patient cohort before matching (Supplementary Table S6). Interaction terms were included to test for effect modification. All statistical analyses were performed using R software, version 4.5.0 (R Foundation for Statistical Computing, Vienna, Austria). All statistical tests were two-tailed, and a *p*-value <0.05 was considered statistically significant.

## Results

### Patient characteristics

**Figure 2.**
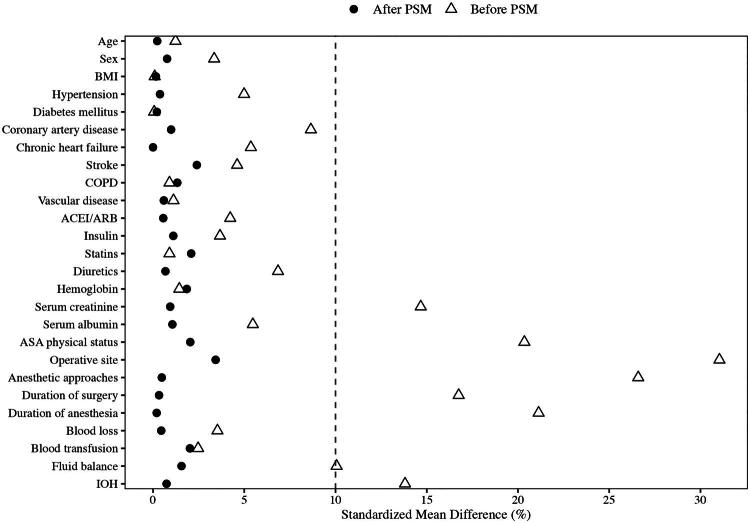
Standardized mean differences (Love plot) of covariates before and after propensity score matching. The dashed line represents the 10% threshold for acceptable balance. SMD: standardized mean difference; PSM: propensity score matching.

A total of 29,497 patients underwent elective major surgery from 2019 to 2022, of which 11,139 were eligible after applying exclusion criteria. Of these, 3,361 patients received NSAIDs (1,577 parecoxib sodium; 1,784 flurbiprofen axetil) and 7,778 did not (Figure 1). Before matching, several baseline characteristics differed between groups: the NSAIDs group had lower baseline serum creatinine, a higher proportion of general anesthesia, longer operative and anesthetic durations, and a higher rate of IOH (Supplementary Table S3). After PS matching, 3,361 pairs were obtained, and all SMDs were <10%, indicating satisfactory covariate balance (Figure 2; Supplementary Table S3).

### Comparison of outcomes

In the overall cohort, the incidence of postoperative AKI was 4.2% (140/3,361) in the NSAIDs group and 4.9% (380/7,778) in the control group (*p* = 0.109). After PS matching, AKI rates were comparable between the matched groups (4.2% [140/3,361] *vs.* 4.2% [140/3,361]; *p* > 0.999). The distribution of AKI stages did not differ significantly between the two groups, either before or after matching. Before matching, the median postoperative hospital stay was significantly longer in the NSAIDs group (6 days [IQR, 5–9] *vs.* 6 days [IQR, 4–8]; *p* < 0.001). Following PS matching, this difference was abolished (6 days [IQR, 5–9] *vs.* 6 days [IQR, 4–9]; *p* = 0.290). Similarly, the incidence of prolonged postoperative hospitalization did not differ significantly after matching (Table 1).

**Table 1.** Outcomes before and after propensity score matching.

Outcomes	Before propensity score matching			After propensity score matching		
	NSAIDs group (*n* = 3,361)	Control group (*n* = 7,778)	*p*-Value	NSAIDs group (*n* = 3,361)	Control group (*n* = 3,361)	*p*-Value
AKI, *n* (%)	140 (4.2%)	380(4.9%)	0.109	140 (4.2%)	140 (4.2%)	>0.999
AKI stages, *n* (%)			0.363			0.830
None	3,221 (95.8%)	7,398 (95.1%)		3,221 (95.8%)	3,221 (95.8%)	
Stage I	121 (3.6%)	327 (4.2%)		121 (3.6%)	126 (3.7%)	
Stage II	10 (0.3%)	23 (0.3%)		10 (0.3%)	7 (0.2%)	
Stage III	9 (0.3%)	30 (0.4%)		9 (0.3%)	7 (0.2%)	
Length of postoperative hospital stay, days	6.0 (5.0, 9.0)	6.0 (4.0, 8.0)	<0.001	6.0 (5.0, 9.0)	6.0 (4.0, 9.0)	0.290
^a^Prolonged postoperative hospital stay, *n* (%)	1,545 (46.0%)	3,173 (40.8%)	<0.001	1,545 (46.0%)	1,546 (46.0%)	>0.999

NSAIDs: nonsteroidal anti-inflammatory drugs; AKI: acute kidney injury.

Values are count (percentage) or median (interquartile range).

^a^ Prolonged postoperative hospital stay was defined as ≥7 days between surgery and discharge.

### Association between NSAIDs and outcomes

**Figure 3.**
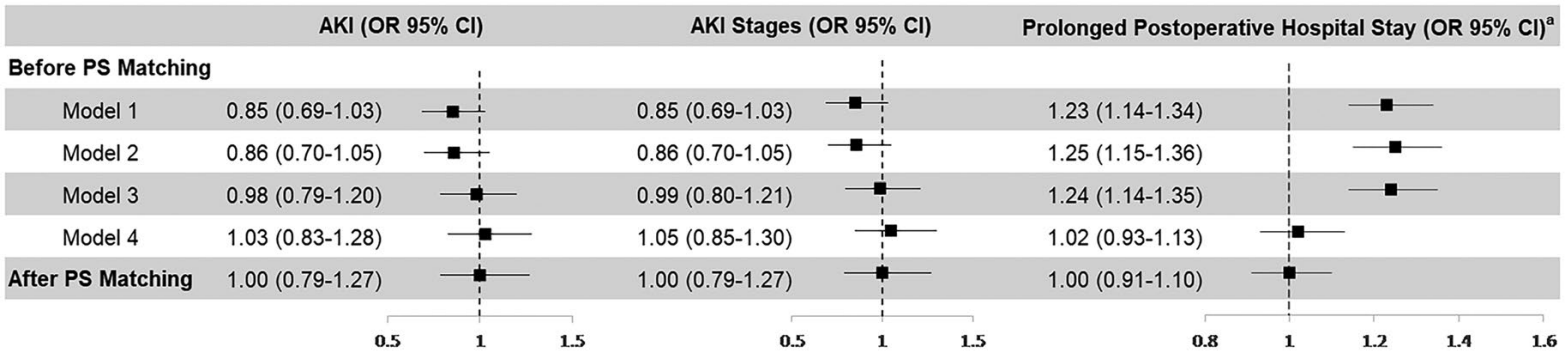
Association between NSAIDs administration and postoperative outcomes. Sequentially adjusted logistic regression models (Models 1–4) were used before propensity score matching; the unadjusted model (Model 1) was used after propensity score matching. Model 1: Unadjusted model. Model 2: Adjusted for age, sex, and BMI. Model 3: Model 2 plus comorbidities (hypertension, diabetes, coronary artery disease, chronic heart failure, stroke, COPD, vascular disease), preoperative medications (ACEI/ARB, statins, insulin, diuretics) and baseline serum creatinine, serum albumin, and serum hemoglobin. Model 4: Model 3 plus ASA physical status, operative site, anesthetic approaches, blood loss, blood transfusion, duration of surgery, duration of anesthesia, IOH, fluid balance. AKI: acute kidney injury; ^a^Prolonged postoperative hospital stay was defined as ≥7 days between surgery and discharge; OR: odds ratio; CI: confidence interval; NSAIDs: nonsteroidal anti-inflammatory drugs; BMI: body mass index; COPD: chronic obstructive pulmonary disease; ACEI: angiotensin-converting enzyme inhibitor; ARB: angiotensin receptor blocker; ASA: American Society of Anesthesiologists; IOH: intraoperative hypotension.

Sequentially adjusted logistic regression analyses were performed with postoperative AKI, AKI stages, and prolonged postoperative hospitalization as dependent variables. In the unadjusted model (Model 1), NSAIDs use showed a trend toward lower odds of AKI before matching (OR 0.85, 95% CI 0.69–1.03; *p* = 0.098) and was associated with higher odds of prolonged stay (OR 1.23, 95% CI 1.14–1.34; *p* < 0.001). However, these associations were attenuated with progressive covariate adjustment. Similar findings were observed after PS matching (Figure 3).

### Stratified analysis by NSAID type and dose-response analysis

Among the 3,361 NSAID recipients, 1,577 received parecoxib sodium (all at 80 mg) and 1,784 received flurbiprofen axetil (742 at ≤50 mg, 1,042 > 50 mg). When stratified by NSAID type with the control group as reference, neither parecoxib sodium (OR 1.24, 95% CI 0.94–1.61; *p* = 0.127) nor flurbiprofen axetil (OR 0.87, 95% CI 0.65–1.15; *p* = 0.339) was significantly associated with AKI in the fully adjusted model before matching. Results were consistent after PS matching. For the combined dose-response analysis, neither low-dose (OR 0.91, 95% CI 0.58–1.35; *p* = 0.641) nor high-dose (OR 1.07, 95% CI 0.85–1.35; *p* = 0.554) NSAIDs were associated with increased AKI risk compared with controls. Results were consistent after PS matching. For flurbiprofen axetil specifically, high-dose subgroups showed no significant association with AKI before and after matching (Table 2).

**Table 2.** Stratified analysis by NSAID type and dose-response analysis.

Group	Before PSM (OR 95% CI)	*p*	After PSM (OR 95% CI)	*p*
Parecoxib *vs.* control	1.24 (0.94–1.61)	0.127	1.25 (0.91–1.69)	0.161
Flurbiprofen *vs.* control	0.87 (0.65–1.15)	0.339	0.86 (0.62–1.18)	0.362
^a^Low dose *vs.* control	0.91 (0.58–1.35)	0.641	0.87 (0.55–1.35)	0.553
^b^High dose (combined) *vs.* control	1.07 (0.85–1.35)	0.554	1.08 (0.83–1.42)	0.565
^c^High dose *vs.* control	0.86 (0.59–1.22)	0.416	0.87 (0.58–1.27)	0.488

PSM: propensity score matching; NSAIDs: nonsteroidal anti-inflammatory drugs; AKI: acute kidney injury.

All values are odds ratios (OR) with 95% confidence intervals (CI) from fully adjusted logistic regression models with the control group as reference.

^a^ Low dose: flurbiprofen axetil ≤50 mg (parecoxib dose-response analysis was not feasible because all patients received 80 mg).

^b^ High dose (combined): parecoxib 80 mg and flurbiprofen axetil >50 mg.

^c^High dose (flurbiprofen axetil only): flurbiprofen axetil >50 mg.

### Subgroup analyses

**Figure 4.**
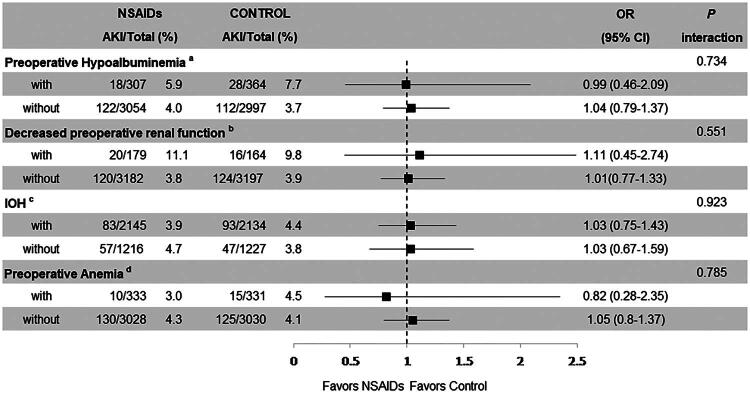
Subgroup analyses assessing the interaction between NSAIDs and preoperative risk factors on the incidence of postoperative AKI in the propensity score-matched cohort. NSAIDs: nonsteroidal anti-inflammatory drugs; AKI: acute kidney injury; OR: odds ratio; CI: confidence interval; ^a^Hypoalbuminemia was defined as a preoperative serum albumin level <3.5 g·dL^−1^; ^b^Decreased preoperative renal function was defined as a preoperative serum creatinine level >1.2 mg·dL^−1^; ^c^Intraoperative hypotension (IOH) was defined as mean arterial pressure (MAP) <65 mmHg; ^d^Preoperative anemia was defined as preoperative serum hemoglobin <100 g·L^−1^.

Subgroup analyses conducted on the propensity score-matched cohort showed no significant interaction between NSAIDs and preoperative hypoalbuminemia (*p* for interaction = 0.734), decreased preoperative renal function (*p* for interaction = 0.551), IOH (*p* for interaction = 0.923), or preoperative anemia (*p* for interaction = 0.785) on the incidence of postoperative AKI. The ORs and 95% CIs of postoperative AKI were not significantly different between NSAIDs and control groups in any stratified subgroup (Figure 4). Subgroup analyses repeated on the entire cohort before matching yielded consistent results (Supplementary Table S6).

### Sensitivity analyses

The missing data report is presented in the Supplementary Table S4. A sensitivity analysis using the complete-case dataset (*n* = 9,385 patients with no missing covariate data) was performed to assess the robustness of the random forest imputation. After PS matching in the complete-case cohort, the results were consistent with the primary analysis, and these findings confirm that the imputation approach did not materially influence the study conclusions (Supplementary Table S5).

## Discussion

In this large retrospective cohort study of 11,139 patients undergoing major noncardiac surgery, we found no significant association between intraoperative NSAIDs use and postoperative AKI. This finding was consistent across multiple analytical approaches, including propensity score matching, sequentially adjusted multivariable models, stratification by NSAID type and dose, and sensitivity analyses using complete-case data.

In contrast to earlier studies that predominantly involved long-term NSAIDs users, our investigation specifically evaluated the renal effects of these agents in the short-term perioperative setting. As essential analgesics in clinical practice, NSAIDs exhibit differing safety profiles based on their mechanisms of action [1]. A secondary analysis of the RELIEF trial suggested that avoiding intraoperative NSAIDs administration might reduce the risk of postoperative AKI [13]. However, accumulating evidence indicates that perioperative NSAIDs use does not increase the risk of AKI in surgical patients. A retrospective analysis of patients undergoing laparoscopic nephrectomy found no association between NSAIDs administered *via* patient-controlled analgesia and postoperative renal dysfunction [27]. A randomized controlled trial demonstrated that parecoxib sodium use during hysterectomy had no adverse impact on postoperative renal function [28]. A prospective propensity-matched study also confirmed no significant association between NSAIDs use and AKI in major gastrointestinal surgery [14]—findings consistent with our results.

The absence of a dose-response relationship further supports the conclusion that perioperative NSAIDs at standard therapeutic doses do not meaningfully increase AKI risk (Table 2). This is clinically reassuring, as both drugs were administered within recommended dose ranges. Subgroup analyses demonstrated no significant interaction between NSAIDs and several established risk factors for AKI, including preoperative hypoalbuminemia, decreased renal function, IOH, and preoperative anemia (Figure 4). These results suggest that the overall null finding is not masked by differential effects in high-risk populations.

Several potential explanations for the null findings merit consideration. First, the intraoperative NSAID exposure in our study was limited to a single administration during surgery, which represents a brief pharmacological exposure unlikely to cause sustained renal prostaglandin inhibition. Second, the majority of our patients had preserved baseline renal function (median creatinine 69–71 μmol·L^−1^), which may buffer against NSAID-induced hemodynamic changes in renal perfusion. Third, the concurrent intravenous fluid administration during surgery may have mitigated any potential nephrotoxic effects through maintenance of renal perfusion. Fourth, residual confounding cannot be excluded despite propensity score matching and multivariable adjustment.

### Limitations

Several limitations should be acknowledged. First, this was a single-center retrospective study from a Chinese academic hospital, which may limit generalizability to other populations and healthcare settings. Second, urine output criteria for AKI diagnosis were not used due to the unavailability of reliable hourly urine output data in our electronic records. This may result in underestimation of AKI incidence, particularly in early or subclinical stages. Third, data on postoperative NSAID use were not available; therefore, we could not assess whether patients in the control group received NSAIDs after surgery, which could bias results toward the null. This represents a potentially important confounder that should be addressed in future studies. Fourth, although we stratified by NSAID type, parecoxib was administered almost exclusively at 80 mg, precluding a meaningful within-drug dose-response analysis. Finally, observational studies can demonstrate associations but cannot establish causality; the impact of different NSAID types, dosages, and duration of exposure on AKI necessitates more rigorously designed prospective studies that integrate medication data throughout the entire perioperative period.

## Conclusion

This propensity score-matched retrospective cohort study found no association between intraoperative NSAIDs use and the incidence or severity of postoperative AKI in patients undergoing major noncardiac surgery.

## Clinical trials registry

Unique identification number for the trial registration: ChiCTR 2300076725.

Registry URL: https://www.chictr.org.cn/index.html.

## Supplementary Material

Supplementary Tables.docx

## Data Availability

The datasets used and/or analyzed during the current study are available from the corresponding author upon reasonable request.
