# Supplementary material for: Impact of intraoperative nonsteroidal anti-inflammatory drugs on acute kidney injury after major noncardiac surgery: a propensity score-matched analysis
Source: Ren Fail. 2026 May 5;48(1):2663247. doi: 10.1080/0886022X.2026.2663247 (PMC13148088; doi:10.1080/0886022X.2026.2663247)
Supplement: Supplementary Tables.docx [file IRNF_A_2663247_SM8829.docx]

**Supplementary Tables**

**Table S1. Modified Johns Hopkins surgical criteria. (P2)**

**Table S2. The International Classification of Disease, Ninth Revision, Clinical Modification Procedure Codes Used for identification of Non-cardiac surgery types. (P3-8)**

**Table S3. Standardized Difference of Covariates Before and After Propensity Score Matching in Patients who Received NSAIDs or not during Elective Major Surgery. (P9-10)**

**Table S4. Missing Data Before Imputation. (P11)**

**Table S5. Sensitivity Analysis: Imputed Data vs. Complete-Case Analysis. (P11)**

**Table S6. Subgroup Analyses Before Propensity Score Matching (Fully Adjusted).(P12)**

| **Table S1. Modified Johns Hopkins surgical criteria** | | | | | | |  |
| --- | --- | --- | --- | --- | --- | --- | --- |
|  | **General** | **Includes** | | | **Excludes** |  |  |
| **Grade I** | Minimal to mild risk independent to anaesthesia | Breast biopsy | | | Open exposure of internal body organs |  |  |
|  |  | Removal of minor skin or subcutaneous lesions | | |  |  |  |
|  | Minimally to moderately invasive procedure | Myringotomy tubes | | | Repair of vascular or neurological structures |  |  |
|  |  | Hysteroscopy | | |  |  |  |
|  | Potential blood loss < 500 mL | Cystoscopy | | | Placement of prosthetic devices |  |  |
|  |  | Vasectomy | | |  |  |  |
|  |  | Circumcision | | | Postoperative monitored care setting |  |  |
|  |  | Fibre‐optic bronchoscopy | | |  |  |  |
|  |  | Diagnostic laparoscopy | | | Open exposure of abdomen, thorax, neck, cranium |  |  |
|  |  | Dilatation and curettage | | |  |  |  |
|  |  | Fallopian tube ligation | | | Resection of major body organs |  |  |
|  |  | Arthroscopy | | |  |  |  |
|  |  | Inguinal hernia repair | | |  |  |  |
|  |  | Laparoscopic lysis of adhesion | | |  |  |  |
|  |  | Tonsillectomy/rhinoplasty | | |  |  |  |
| **Grade II** | Moderately to significantly invasive procedures | Thyroidectomy | | | Open thoracic or intracranial procedure |  |  |
|  |  | Hysterectomy | | |  |  |  |
|  | Potential blood loss 500 to 1500 mL | Myomectomy | | | Major vascular repair (e.g. aortofemoral bypass) |  |  |
|  |  | Cystectomy | | |  |  |  |
|  | Moderate risk to patient independent of anaesthesia | Cholecystectomy | | | Planned postoperative monitored care setting (ICU, ACU) |  |  |
|  |  | Laminectomy | | |  |  |  |
|  |  | Hip/knee replacement | | |  |  |  |
|  |  | Nephrectomy | | |  |  |  |
|  |  | Major laparoscopic procedures | | |  |  |  |
|  |  | Resection/reconstructive surgery of the digestive tract | |  | |  |  |
|  |  |  |  |  | |  |  |
| **Grade III** | Highly invasive procedure | Major orthopaedic-spinal reconstruction | |  | |  |  |
|  |  | Major reconstruction of the gastrointestinal tract | | |  | | |
|  | Potential blood loss > 1500 mL | Major genitourinary surgery (e.g. radical | | |  |  |  |
|  |  | retropubic prostatectomy) | | |  |  |  |
|  | Major to critical risk to patient independent of anaesthesia | Major vascular repair without postoperative ICU stay | | |  |  |  |
|  |  | Cardiothoracic procedure | | |  |  |  |
|  |  | Intracranial procedure | | |  |  |  |
|  | Usual postoperative ICU stay with invasive monitoring | Major procedure on the oropharynx | | |  |  |  |
|  |  | Major vascular, skeletal, neurological repair |  | | |  |  |

PACU, post anaesthesia care unit; ICU, intensive care unit

| **Table S2. The International Classification of Disease, Ninth Revision, Clinical Modification Procedure Codes Used for identification of Non-cardiac surgery types** | | |
| --- | --- | --- |
| **surgical site** | **ICD-9CM procedure code** | **Description** |
| abdominal | 1732 | Laparoscopic cecectomy |
|  | 1733 | Laparoscopic right hemicolectomy |
|  | 1734 | Laparoscopic resection of transverse colon |
|  | 1735 | Laparoscopic left hemicolectomy |
|  | 1736 | Laparoscopic sigmoidectomy |
|  | 1739 | Other laparoscopic partial excision of large intestine |
|  | 415 | Total splenectomy |
|  | 4342 | Local excision of other lesion or tissue of stomach |
|  | 435 | Partial gastrectomy with anastomosis to esophagus |
|  | 436 | Partial gastrectomy with anastomosis to duodenum |
|  | 437 | Partial gastrectomy with anastomosis to jejunum |
|  | 4382 | Laparoscopic vertical (sleeve) gastrectomy |
|  | 4389 | Open and other partial gastrectomy |
|  | 4399 | Other total gastrectomy |
|  | 4438 | Laparoscopic gastroenterostomy |
|  | 4439 | Other gastroenterostomy without gastrectomy |
|  | 4463 | Closure of other gastric fistula |
|  | 4531 | Other local excision of lesion of duodenum |
|  | 4562 | Other partial resection of small intestine |
|  | 4563 | Total removal of small intestine |
|  | 4572 | Open and other cecectomy |
|  | 4573 | Open and other right hemicolectomy |
|  | 4574 | Open and other resection of transverse colon |
|  | 4575 | Open and other left hemicolectomy |
|  | 4576 | Open and other sigmoidectomy |
|  | 4579 | Other and unspecified partial excision of large intestine |
|  | 4581 | Laparoscopic total intra-abdominal colectomy |
|  | 4582 | Open total intra-abdominal colectomy |
|  | 4611 | Temporary colostomy |
|  | 4613 | Permanent colostomy |
|  | 4651 | Closure of stoma of small intestine |
|  | 4652 | Closure of stoma of large intestine |
|  | 4701 | Laparoscopic appendectomy |
|  | 4709 | Other appendectomy |
|  | 4851 | Laparoscopic abdominoperineal resection of the rectum |
|  | 4852 | Open abdominoperineal resection of the rectum |
|  | 4862 | Anterior resection of rectum with synchronous colostomy |
|  | 4863 | Other anterior resection of rectum |
|  | 4869 | Other resection of rectum |
|  | 500 | Hepatotomy |
|  | 5022 | Partial hepatectomy |
|  | 5023 | Open ablation of liver lesion or tissue |
|  | 5025 | Laparoscopic ablation of liver lesion or tissue |
|  | 5029 | Other destruction of lesion of liver |
|  | 503 | Lobectomy of liver |
|  | 5122 | Cholecystectomy |
|  | 5123 | Laparoscopic cholecystectomy |
|  | 5124 | Laparoscopic partial cholecystectomy |
|  | 5141 | Common duct exploration for removal of calculus |
|  | 5151 | Exploration of common duct |
|  | 5163 | Other excision of common duct |
|  | 5164 | Endoscopic excision or destruction of lesion of biliary ducts or sphincter of Oddi |
|  | 5169 | Excision of other bile duct |
|  | 5188 | Endoscopic removal of stone(s) from biliary tract |
|  | 5251 | Proximal pancreatectomy |
|  | 5252 | Distal pancreatectomy |
|  | 5253 | Radical subtotal pancreatectomy |
|  | 5259 | Other partial pancreatectomy |
|  | 526 | Total pancreatectomy |
|  | 527 | Radical pancreaticoduodenectomy |
|  | 5411 | Exploratory laparotomy |
|  | 543 | Excision or destruction of lesion or tissue of abdominal wall or umbilicus |
|  | 544 | Excision or destruction of peritoneal tissue |
|  | 5459 | Other lysis of peritoneal adhesions |
|  | 5499 | Other operations of abdominal region |
| Orthopaedic | 0071 | Revision of hip replacement, acetabular component |
|  | 0301 | Removal of foreign body from spinal canal |
|  | 0309 | Other exploration and decompression of spinal canal |
|  | 034 | Excision or destruction of lesion of spinal cord or spinal meninges |
|  | 0353 | Repair of vertebral fracture |
|  | 036 | Lysis of adhesions of spinal cord and nerve roots |
|  | 7705 | Sequestrectomy, femur |
|  | 7707 | Sequestrectomy, tibia and fibula |
|  | 7725 | Wedge osteotomy, femur |
|  | 7727 | Wedge osteotomy, tibia and fibula |
|  | 7729 | Wedge osteotomy, other bones |
|  | 7765 | Local excision of lesion or tissue of bone, femur |
|  | 7767 | Local excision of lesion or tissue of bone, tibia and fibula |
|  | 7769 | Local excision of lesion or tissue of bone, other bones |
|  | 7785 | Other partial ostectomy, femur |
|  | 7787 | Other partial ostectomy, tibia and fibula |
|  | 7789 | Other partial ostectomy, other bones |
|  | 7812 | Application of external fixator device, humerus |
|  | 7815 | Application of external fixator device, femur |
|  | 7817 | Application of external fixator device, tibia and fibula |
|  | 7835 | Limb lengthening procedures, femur |
|  | 7837 | Limb lengthening procedures, tibia and fibula |
|  | 7841 | Other repair or plastic operations on bone, scapula, clavicle, and thorax [ribs and sternum] |
|  | 7845 | Other repair or plastic operations on bone, femur |
|  | 7855 | Internal fixation of bone without fracture reduction, femur |
|  | 7857 | Internal fixation of bone without fracture reduction, tibia and fibula |
|  | 7859 | Internal fixation of bone without fracture reduction, other bones |
|  | 7865 | Removal of implanted devices from bone, femur |
|  | 7869 | Removal of implanted devices from bone, other bones |
|  | 7905 | Closed reduction of fracture without internal fixation, femur |
|  | 7915 | Closed reduction of fracture with internal fixation, femur |
|  | 7925 | Open reduction of fracture without internal fixation, femur |
|  | 7931 | Open reduction of fracture with internal fixation, humerus |
|  | 7932 | Open reduction of fracture with internal fixation, radius and ulna |
|  | 7935 | Open reduction of fracture with internal fixation, femur |
|  | 7936 | Open reduction of fracture with internal fixation, tibia and fibula |
|  | 7937 | Open reduction of fracture with internal fixation, tarsals and metatarsals |
|  | 7939 | Open reduction of fracture with internal fixation, other specified bone |
|  | 7985 | Open reduction of dislocation of hip |
|  | 7987 | Open reduction of dislocation of ankle |
|  | 7989 | Open reduction of dislocation of other specified sites |
|  | 8046 | Division of joint capsule, ligament, or cartilage, knee |
|  | 8051 | Excision of intervertebral disc |
|  | 8059 | Other destruction of intervertebral disc |
|  | 8099 | Other excision of joint, other specified sites |
|  | 8101 | Atlas-axis spinal fusion |
|  | 8102 | Other cervical fusion of the anterior column, anterior technique |
|  | 8103 | Other cervical fusion of the posterior column, posterior technique |
|  | 8104 | Dorsal and dorsolumbar fusion of the anterior column, anterior technique |
|  | 8105 | Dorsal and dorsolumbar fusion of the posterior column, posterior technique |
|  | 8106 | Lumbar and lumbosacral fusion of the anterior column, anterior technique |
|  | 8107 | Lumbar and lumbosacral fusion of the posterior column, posterior technique |
|  | 8108 | Lumbar and lumbosacral fusion of the anterior column, posterior technique |
|  | 8122 | Arthrodesis of knee |
|  | 8131 | Refusion of atlas-axis spine |
|  | 8135 | Refusion of dorsal and dorsolumbar spine, posterior column, posterior technique |
|  | 8138 | Refusion of lumbar and lumbosacral spine, anterior column, posterior technique |
|  | 8151 | Total hip replacement |
|  | 8152 | Partial hip replacement |
|  | 8153 | Revision of hip replacement, not otherwise specified |
|  | 8154 | Total knee replacement |
|  | 8405 | Amputation through forearm |
|  | 8410 | Lower limb amputation, not otherwise specified |
|  | 8411 | Amputation of toe |
|  | 8412 | Amputation through foot |
|  | 8415 | Other amputation below knee |
|  | 8416 | Disarticulation of knee |
|  | 8417 | Amputation above knee |
|  | 8418 | Disarticulation of hip |
| genitourinary | 576 | Partial cystectomy |
|  | 5771 | Radical cystectomy |
|  | 5779 | Other total cystectomy |
|  | 605 | Radical prostatectomy |
|  | 6062 | Perineal prostatectomy |
|  | 6069 | Other prostatectomy |
|  | 631 | Excision of varicocele and hydrocele of spermatic cord |
|  | 6529 | Other local excision or destruction of ovary |
|  | 6531 | Laparoscopic unilateral oophorectomy |
|  | 6539 | Other unilateral oophorectomy |
|  | 6541 | Laparoscopic unilateral salpingo-oophorectomy |
|  | 6549 | Other unilateral salpingo-oophorectomy |
|  | 6551 | Other removal of both ovaries at same operative episode |
|  | 6553 | Laparoscopic removal of both ovaries at same operative episode |
|  | 6561 | Other removal of both ovaries and tubes at same operative episode |
|  | 6563 | Laparoscopic removal of both ovaries and tubes at same operative episode |
|  | 664 | Total unilateral salpingectomy |
|  | 6839 | Other and unspecified subtotal abdominal hysterectomy |
|  | 6841 | Laparoscopic total abdominal hysterectomy |
|  | 6849 | Other and unspecified total abdominal hysterectomy |
|  | 6861 | Laparoscopic radical abdominal hysterectomy |
|  | 6869 | Other and unspecified radical abdominal hysterectomy |
|  | 688 | Pelvic evisceration |
|  | 6919 | Other excision or destruction of uterus and supporting structures |
|  | 7077 | Vaginal suspension and fixation |
| Head and neck | 062 | Unilateral thyroid lobectomy |
|  | 0639 | Other partial thyroidectomy |
|  | 064 | Complete thyroidectomy |
|  | 2231 | Radical maxillary antrot |
|  | 2431 | Excision of lesion or tissue of gum |
|  | 252 | Partial glossectomy |
|  | 254 | Radical glossectomy |
|  | 2749 | Other excision of mouth |
|  | 2799 | Other operations on oral cavity |
|  | 2933 | Pharyngectomy (partial) |
|  | 301 | Hemilaryngectomy |
|  | 3029 | Other partial laryngectomy |
|  | 303 | Complete laryngectomy |
|  | 304 | Radical laryngectomy |
|  | 7631 | Partial mandibulectomy |
| thoracic | 3220 | Thoracoscopic excision of lesion or tissue of lung |
|  | 3221 | Plication of emphysematous bleb |
|  | 3229 | Other local excision or destruction of lesion or tissue of lung |
|  | 3230 | Thoracoscopic segmental resection of lung |
|  | 3241 | Thoracoscopic lobectomy of lung |
|  | 3249 | Other lobectomy of lung |
|  | 3250 | Thoracoscopic pneumonectomy |
|  | 3259 | Other and unspecified pneumonectomy |
|  | 3409 | Other incision of pleura |
|  | 343 | Excision or destruction of lesion or tissue of mediastinum |
|  | 3451 | Decortication of lung |
|  | 3459 | Other excision of pleura |
|  | 4209 | Other incision of esophagus |
|  | 4232 | Local excision of other lesion or tissue of esophagus |
|  | 4233 | Endoscopic excision or destruction of lesion or tissue of esophagus |
|  | 4241 | Partial esophagectomy |
|  | 4242 | Total esophagectomy |
|  | 4251 | Intrathoracic esophagoesophagostomy |
|  | 4258 | Intrathoracic esophageal anastomosis with other interposition |
| vascular | 3806 | Incision of vessel, abdominal arteries |
|  | 3838 | Resection of vessel with anastomosis, lower limb arteries |
|  | 3844 | Resection of vessel with replacement, aorta, abdominal |
|  | 3845 | Resection of vessel with replacement, thoracic vessels |
|  | 3846 | Resection of vessel with replacement, abdominal arteries |
|  | 3848 | Resection of vessel with replacement, lower limb arteries |
|  | 3866 | Other excision of vessels, abdominal arteries |
|  | 3868 | Other excision of vessels, lower limb arteries |
|  | 3925 | Aorta-iliac-femoral bypass |
|  | 3926 | Other intra-abdominal vascular shunt or bypass |
|  | 3929 | Other (peripheral) vascular shunt or bypass |
|  | 3949 | Other revision of vascular procedure |
|  | 3953 | Repair of arteriovenous fistula |
|  | 3971 | Endovascular implantation of other graft in abdominal aorta |
|  | 3973 | Endovascular implantation of graft in thoracic aorta |
|  | 3978 | Endovascular implantation of branching or fenestrated graft(s) in aorta |
|  | 3979 | Other endovascular procedures on other vessels |

**Supplementary Table S3.** **Standardized Difference of Covariates** **Before and After Propensity Score Matching in Patients who Received NSAIDs or not during Elective Major Surgery.**

|  |  | **Before Propensity Score Matching** | | | **After Propensity Score Matching** | | |
| --- | --- | --- | --- | --- | --- | --- | --- |
| **Characteristic** |  | **NSAIDs Group** | **Control Group** | **Strandardized** | **NSAIDs Group** | **Control Group** | **Strandardized** |
|  |  | **(n=3361)** | **(n=7778)** | **Difference %** | **(n=3361)** | **(n=3361)** | **Difference %** |
| Age, y |  | 54.0 (45.0, 63.0) | 54.0 (43.0, 63.0) | 1.23 | 54.0 (45.0, 63.0) | 54.0 (44.0, 63.0) | 0.28 |
| Sex, male, n (%) |  | 1635 (48.6%) | 3914 (50.3%) | 3.35 | 1635 (48.6%) | 1618 (48.1%) | 1.01 |
| BMI, kg·m⁻² |  | 23.2 (21.1, 25.5) | 23.2 (21.1, 25.5) | 0.07 | 23.2 (21.1, 25.5) | 23.2 (21.0, 25.4) | 1.06 |
| Coexisting medical conditions, n (%) | | | | | | | |
| Hypertension |  | 644 (19.2%) | 1646 (21.2%) | 4.99 | 644 (19.2%) | 656 (19.5%) | 0.9 |
| Diabetes mellitus |  | 282 (8.4%) | 654 (8.4%) | 0.06 | 282 (8.4%) | 296 (8.8%) | 1.49 |
| Coronary artery disease |  | 47 (1.4%) | 203 (2.6%) | 8.65 | 47 (1.4%) | 46 (1.4%) | 0.25 |
| Chronic heart failure |  | 1 (<0.1%) | 17 (0.2%) | 5.36 | 1 (<0.1%) | 1 (<0.1%) | 0 |
| Stroke |  | 47 (1.4%) | 155 (2.0%) | 4.61 | 47 (1.4%) | 51 (1.5%) | 0.99 |
| COPD |  | 25 (0.7%) | 64 (0.8%) | 0.9 | 25 (0.7%) | 27 (0.8%) | 0.68 |
| Vascular disease |  | 8 (0.2%) | 23 (0.3%) | 1.12 | 8 (0.2%) | 10 (0.3%) | 1.15 |
| Preoperative medications, n (%) | | | | | | | |
| Chronic use of ACEI/ARB |  | 161 (4.8%) | 446 (5.7%) | 4.23 | 161 (4.8%) | 164 (4.9%) | 0.42 |
| Insulin |  | 331 (9.8%) | 683 (8.8%) | 3.67 | 331 (9.8%) | 334 (9.9%) | 0.3 |
| Statins |  | 169 (5.0%) | 376 (4.8%) | 0.9 | 169 (5.0%) | 162 (4.8%) | 0.96 |
| diuretics |  | 103 (3.1%) | 339 (4.4%) | 6.85 | 103 (3.1%) | 106 (3.2%) | 0.51 |
| Preoperative laboratory test | | | | | | | |
| Serum hemoglobin, g·L⁻¹ |  | 128.0(116.0, 140.0) | 129.0 (115.0, 141.0) | 1.48 | 128.0 (116.0, 140.0) | 128.0(114.0, 140.0) | 2.57 |
| Serum creatinine, μmol·L⁻¹ |  | 69.0 (58.0, 83.0) | 71.0 (59.0, 85.0) | 14.67 | 69.0 (58.0, 83.0) | 69.0 (58.0, 82.0) | 0.27 |
| Serum albumin, g·L⁻¹ |  | 39.9 (37.4, 42.8) | 39.9 (37.3, 42.7) | 5.47 | 39.9 (37.4, 42.8) | 39.9 (37.4, 42.8) | 1.64 |
| ASA physical status, n (%) |  |  |  | 20.35 |  |  | 1.14 |
| Ⅰ |  | 699 (20.8%) | 1314 (16.9%) |  | 699 (20.8%) | 696 (20.7%) |  |
| Ⅱ |  | 2,472 (73.5%) | 5663 (72.8%) |  | 2472 (73.5%) | 2469 (73.5%) |  |
| Ⅲ |  | 187 (5.6%) | 732 (9.4%) |  | 187 (5.6%) | 192 (5.7%) |  |
| Ⅳ |  | 3 (0.1%) | 69 (0.9%) |  | 3 (0.1%) | 4 (0.1%) |  |
| Operative site, n (%) |  |  |  | 31.04 |  |  | 3.41 |
| Abdominal |  | 1259 (37.5%) | 1883 (24.2%) |  | 1259 (37.5%) | 1276 (38.0%) |  |
| Orthopedic |  | 419 (12.5%) | 1269 (16.3%) |  | 419 (12.5%) | 434 (12.9%) |  |
| Genitourinary |  | 1014 (30.2%) | 2799 (36.0%) |  | 1014 (30.2%) | 989 (29.4%) |  |
| Head and neck |  | 67 (2.0%) | 261 (3.4%) |  | 67 (2.0%) | 74 (2.2%) |  |
| Thoracic |  | 595 (17.7%) | 1497 (19.2%) |  | 595 (17.7%) | 584 (17.4%) |  |
| Vascular |  | 7 (0.2%) | 69 (0.9%) |  | 7 (0.2%) | 4 (0.1%) |  |
| Anesthetic Approaches, n (%) |  |  |  | 26.59 |  |  | 0.12 |
| General |  | 3146 (93.6%) | 6654 (85.5%) |  | 3146 (93.6%) | 3145 (93.6%) |  |
| Intraspinal |  | 215 (6.4%) | 1124 (14.5%) |  | 215 (6.4%) | 216 (6.4%) |  |
| Duration of surgery, min |  | 150.0(105.0, 207.0) | 130.0 (84.0, 193.0) | 16.75 | 150.0 (105.0, 207.0) | 146.0 (97.0, 215.0) | 0.95 |
| Duration of anesthesia, min |  | 269.0(210.0, 341.0) | 240.0 (176.0, 322.0) | 21.13 | 269.0 (210.0, 341.0) | 265.0(200.0, 353.0) | 0.73 |
| Blood loss, mL·kg⁻¹ |  | 0.8 (0.3, 1.7) | 0.8 (0.2, 1.8) | 3.58 | 0.8 (0.3, 1.7) | 0.8 (0.3, 1.7) | 1.27 |
| Blood transfusion, n (%) |  | 280 (8.3%) | 702 (9.0%) | 2.47 | 280 (8.3%) | 278 (8.3%) | 0.22 |
| Fluid Balance, mL·kg⁻¹ |  | 18.9 (12.9, 26.5) | 17.0 (11.1, 24.8) | 9.98 | 18.9 (12.9, 26.5) | 18.5 (12.5, 27.2) | 1.5 |
| IOH, n (%) |  | 2145(63.8%) | 4440(57.1%) | 13.81 | 2145(63.8%) | 2134(63.5%) | 0.68 |

Values are count (percentage) or median (interquartile range); NSAIDs, nonsteroidal anti-inflammatory drugs; BMI, body mass index; COPD, chronic obstructive pulmonary disease; ACEI, angiotensin-converting enzyme inhibitor; ARB, angiotensin receptor blocker; ASA, American Society of Anesthesiologists; IOH, intraoperative hypotension

**Supplementary Table S4.** **Missing Data Before Imputation (N = 11,139).** **Values are n (%).**

| Variable | Overall | NSAIDs | Control |
| --- | --- | --- | --- |
| Height | 180 (1.6%) | 26 (0.8%) | 154 (2.0%) |
| Weight | 216 (1.9%) | 37 (1.1%) | 179 (2.3%) |
| BMI | 234 (2.1%) | 42 (1.2%) | 192 (2.5%) |
| Serum albumin | 138 (1.2%) | 47 (1.4%) | 91 (1.2%) |
| Hemoglobin | 144 (1.3%) | 48 (1.4%) | 96 (1.2%) |
| Blood loss | 1,588 (14.3%) | 397 (11.8%) | 1,191 (15.3%) |
| Fluid balance | 223 (2.0%) | 38 (1.1%) | 185 (2.4%) |

**Supplementary Table S5**. **Sensitivity Analysis: Imputed Data vs. Complete-Case Analysis.**

| Analysis | N | AKI  OR (95% CI) | *P* | | AKI Stage  OR (95% CI) | *P* | Prolonged Stay  OR (95% CI) | *P* |
| --- | --- | --- | --- | --- | --- | --- | --- | --- |
| Imputed - Before PSM (Fully Adjusted) | 11,139 | 1.03 (0.83-1.28) | | 0.753 | 1.05 (0.85-1.30) | 0.664 | 1.02 (0.93-1.13) | 0.638 |
| Complete Case - Before PSM (Fully Adjusted) | 9,385 | 1.11 (0.88-1.4) | | 0.365 | 1.13 (0.9-1.43) | 0.291 | 1.04 (0.93-1.16) | 0.476 |
| Imputed - After PSM | 6,722 | 1.00 (0.79-1.27) | | 1.000 | 1.00 (0.79-1.27) | 0.990 | 1.00 (0.91-1.10) | 0.980 |
| Complete Case - After PSM | 5,818 | 0.99 (0.77-1.28) | | 0.948 | 0.99 (0.77-1.28) | 0.966 | 1.03 (0.93-1.14) | 0.545 |

OR, odds ratio; CI, confidence interval; AKI, acute kidney injury; PSM, propensity score matching.

**Supplementary Table S6. Subgroup Analyses Before Propensity Score Matching (Fully Adjusted).**

| Subgroup | Level | N | AKI | OR_CI | P_interaction |
| --- | --- | --- | --- | --- | --- |
| Preoperative Hypoalbuminemia | without | 9916 | 412 | 1.07 (0.85-1.34) | 0.703 |
|  | with | 1223 | 108 | 0.87 (0.47-1.57) | 0.703 |
| Decreased preoperative renal function | without | 10319 | 397 | 1.07 (0.85-1.35) | 0.492 |
|  | with | 820 | 123 | 0.82 (0.45-1.46) | 0.492 |
| IOH | without | 4554 | 210 | 1.06 (0.75-1.47) | 0.685 |
|  | with | 6585 | 310 | 1.03 (0.78-1.36) | 0.685 |
| Preoperative Anemia | without | 10019 | 448 | 1.06 (0.85-1.32) | 0.352 |
|  | with | 1120 | 72 | 0.74 (0.32-1.58) | 0.352 |

OR, odds ratio; CI, confidence interval; AKI, acute kidney injury; IOH, intraoperative hypotension. Hypoalbuminemia: serum albumin <3.5 g·dL⁻¹; Decreased preoperative renal function: serum creatinine >1.2 mg·dL⁻¹; IOH: mean arterial pressure <65 mmHg; Anemia: hemoglobin <100 g·L⁻¹."
